# Detecting differentially methylated loci for Illumina Array methylation data based on human ovarian cancer data

**DOI:** 10.1186/1755-8794-6-S1-S9

**Published:** 2013-01-23

**Authors:** Zhongxue Chen, Hanwen Huang, Jianzhong Liu, Hon Keung Tony Ng, Saralees Nadarajah, Xudong Huang, Youping Deng

**Affiliations:** 1 Department of Epidemiology and Biostatistics, School of Public Health, Indiana University Bloomington, 1025 E. 7th Street, Bloomington, IN 47405, USA; 2 Center for Clinical and Translational Sciences, The University of Texas Health Science Center at Houston, Houston, TX 77030, USA; 3 Chem21 Group, Inc, 1780 Wilson Drive, Lake Forest, IL 60045, USA; 4 Department of Statistical Science, Southern Methodist University, Dallas, TX 75275, USA; 5 School of Mathematics, University of Manchester, Manchester, M13 9PL, UK; 6 Neurochemistry Laboratory, Department of Psychiatry, Massachusetts General Hospital and Harvard Medical School, Charlestown, MA 02129, USA; 7 Rush University Cancer Center, Department of Internal Medicine and Biochemistry, Rush University Medical Center, Chicago, IL 60612, USA

## Abstract

**Background:**

It is well known that DNA methylation, as an epigenetic factor, has an important effect on gene expression and disease development. Detecting differentially methylated loci under different conditions, such as cancer types or treatments, is of great interest in current research as it is important in cancer diagnosis and classification. However, inappropriate testing approaches can result in large false positives and/or false negatives. Appropriate and powerful statistical methods are desirable but very limited in the literature.

**Results:**

In this paper, we propose a nonparametric method to detect differentially methylated loci under multiple conditions for Illumina Array Methylation data. We compare the new method with other methods using simulated and real data. Our study shows that the proposed one outperforms other methods considered in this paper.

**Conclusions:**

Due to the unique feature of the Illumina Array Methylation data, commonly used statistical tests will lose power or give misleading results. Therefore, appropriate statistical methods are crucial for this type of data. Powerful statistical approaches remain to be developed.

**Availability:**

R codes are available upon request.

## Background

It is well known that DNA methylation has important effects on transcriptional regulation, chromosomal stability, genomic imprinting, and X-inactivation [1,2]. It has been also shown to be associated with many human diseases, such as various types of cancer [3-11].

With the advances of BeadArray technology, genome-wide high-throughput methylation data can be easily generated by Illumina GoldenGate and Infinium Methylation Assays. After preprocessing steps, such as background correction and normalization, are applied to the raw fluorescent intensities, for each locus, from about 30 replicates in the same array a summarized *β*-value is generated as follows: $\frac{\text{max}\left\{M,0\right\}}{\text{max}\left\{M,0\right\}+\text{max}\left\{U,0\right\}+100}$, where *M *is the average signal from a methylated allele while *U *is that from unmethylated allele. The *β *-values are continuous numbers between 0 and 1, with 0 stands for totally unmethylated and 1 for completely methylated.

It has been shown that the *β *-value is rarely normally distributed [9,12,13]. Therefore the commonly used t-test for case control designs or ANOVA for multiple conditions are not the most powerful approaches when detecting differentially methylated loci. Observing this, Wang has proposed a model-based likelihood ratio test to detect differentially methylated loci for case and control data under the assumption that the *β *-value follows a three-component normal-uniform distribution [9]. Wang showed that for some situations, their proposed test was better than the simple t-test based on simulation studies.

However, in their method, Wang did not consider the effect of age, which has been shown highly associated with methylation [14,15]. Noticing the importance of age effect, one may use a linear regression with age included as a covariate when analyze methylation data with multiple conditions, such as cancer types. However, the underlying assumption of equal variances may not be satisfied [12]. Therefore the commonly used linear regression method may not be appropriate.

In this paper, we consider methylation data with multiple conditions and propose a nonparametric method which incorporates the age effect in a way through the idea of combining p-values from independent tests [12,16,17]. More specifically, we first group subjects into several age groups based on their age; then for each age group, a nonparametric Kruskal-Wallis test is conducted for the given locus and the p-value is recorded. An overall p-value for that locus will be estimated through combining the p-values from all age groups. Using a real methylation data with three conditions and a simulation study, we show that the proposed test is more powerful than other methods, including linear regression.

## Method

### Proposed method

Assume there are *K *conditions and *G *age groups. For each age group *g (g = 1,2,...,G)*, we apply the nonparametric Kruskal-Wallis test and obtain a p-value $p_{g}^{KW}$, then the overall p-value can be estimated by Fisher test [18]:

(1) $$p_{KW}=\chi_{df=2G}^{2}\left(\chi^{2}>-2\overset{G}{\underset{g=1}{\sum }}\text{log}\left(p_{g}^{KW}\right)\right)$$

### Combined ANOVA test

Similarly, we can use ANOVA to replace KW test for each age group and obtain an overall p-value with $p_{g}^{KW}$ being replaced by the p-value $p_{g}^{ANOVA}$ from ANOVA test:

(2) $$p_{ANOVA}=\chi_{df=2G}^{2}\left(\chi^{2}>-2\overset{G}{\underset{g=1}{\sum }}\text{log}\left(p_{g}^{ANOVA}\right)\right)$$

### Combined median test

Another nonparametric test is median test using the following statistic for each age group:

$M=4\overset{K}{\underset{k=1}{\sum }}\frac{{\left(A_{k}-n_{k}/2\right)}^{2}}{n_{k}}$, where *A_k _*is the number of times that the ranks of individual observations from group k which excess the median from the pooled data, and *n_k _*is the sample size of group *k*. When the sample sizes are large, under the null hypothesis that all samples have the same median, the statistic M has a chi-square distribution with K-1 degrees of freedom. The overall p-value from the combined median test can be calculated:

(3) $$p_{Median}=\chi_{df=2G}^{2}\left(\chi^{2}>-2\overset{G}{\underset{g=1}{\sum }}\text{log}\left(p_{g}^{Median}\right)\right)$$

### Combined welch test

We also consider the nonparametric Welch test. For each age group, we have the test statistic [19]:

$$W=\frac{\overset{K}{\underset{k=1}{\sum }}w_{k}{\left({\bar{\bar{x}}}_{k}-\hat{\mu }\right)}^{2}/\left(K-1\right)}{1+\left[2\left(K-2\right)/\left(K^{2}-2\right)\right]\overset{K}{\underset{k=1}{\sum }}h_{k}},$$

where $w_{k}=n_{k}/s_{k}^{2},\;\hat{\mu }\text{ = }\overset{K}{\underset{k=1}{\sum }}w_{k}{\bar{x}}_{k}/w,\text{ w = }\overset{K}{\underset{k=1}{\sum }}w_{k},\;{\text{h}}_{k}=\left(1-w_{k}/w^{2}\right)/\left(n_{k}-1\right)$.

Under the null hypothesis, the statistic W is asymptotically distributed as F-distribution with *K*-1 and $\text{f = (}{\text{K}}^{2}\text{ - 1)/(3}\overset{K}{\underset{k=1}{\sum }}h_{k}\text{)}$ degrees of freedom. Welch test is an improvement of the Cochran test [20] which usually has inflated type I error rate especially for small sample sizes [19,21]. The overall p-value from the combined Welch test is:

(4) $$p_{Welch}=\chi_{df=2G}^{2}\left(\chi^{2}>-2\overset{G}{\underset{g=1}{\sum }}\text{log}\left(p_{g}^{Welch}\right)\right)$$

### Methods for combining p-values

Besides the Fisher method mentioned above, we also consider Z-test to combine p-values from independent tests. First we calculated the weighted Z statistic using individual p-values from each age group: $Z=\overset{G}{\underset{g=1}{\sum }}n_{g}\Phi^{-1}\left(1-p_{g}\right)/\overset{G}{\underset{g=1}{\sum }}n_{g}^{2}$, where *n_g _*is the total sample size in age group *g *and Φ is the cumulative distribution function (CDF) of the standard normal distribution. It is easy to see that this statistic has standard normal distribution under the null hypothesis. The overall p-value is calculated by 1- Φ(Z). Note that here we use one-sided test to obtain the overall p-value.

### Simulation settings

To compare each method applied to an individual age group, we simulate *β *-value for three treatment groups based on beta distribution with parameters *a *and *b*, beta (*a,b*), and truncated normal distribution on (0,1) with parameters μ, σ^2^, TN(μ, σ^2^). We assume the sample sizes (denoted as *s *in Tables 1, 2 for the simulation results) for the three treatments are either balanced: *s *= 30 for each, or non-balanced: *s *= 20, 30, and 40. First we compare the estimated type I error rates with the given significance level of 0.05 under the null hypothesis of no differences among treatment groups. Then we compare the empirical powers from each method under various situations. The empirical power is the proportion of rejected null hypothesis to the number of replicates.

**Table 1:** Estimated type I error rates at significance level 0.05 with 10000 replicates.

Distribution (sample sizes, parameters)	ANOVA	median	Welch	KW
Beta (*s *= 30,30,30, *a *= 1,1,1, *b *= 2,2,2)	0.048	0.040	0.052	0.047
Beta (*s *= 30,30,30, *a *= 1,1,1, *b *= 10,10,10)	0.052	0.044	0.053	0.051
Beta (*s *= 30,30,30, *a *= 10,10,10, *b *= 1,1,1)	0.047	0.044	0.052	0.048
Beta (*s *= 30,30,30, *a *= 10,10,10, *b *= 10,10,10)	0.045	0.045	0.047	0.046
Beta (*s *= 20,30,40, *a *= 1,1,1, *b *= 2,2,2)	0.053	0.052	0.050	0.053
Beta (*s *= 20,30,40, *a *= 1,1,1, *b *= 10,10,10)	0.049	0.049	0.054	0.048
Beta (*s *= 20,30,40, *a *= 10,10,10, *b *= 1,1,1)	0.045	0.049	0.056	0.044
Beta (*s *= 20,30,40, *a *= 10,10,10, *b *= 10,10,10)	0.050	0.051	0.043	0.052
TN (*s *= 30,30,30, μ = 0.5,0.5, 0.5, σ^2 ^= 0.1,0.1,0.1)	0.050	0.044	0.053	0.045
TN(*s *= 30,30,30, μ = 0.5, 0.5, 0.5, σ^2 ^= 0.1,0.2,0.3)	0.053	0.067	0.047	0.053
TN (*s *= 20,30,40, μ = 0.5, 0.5, 0.5, σ^2 ^= 0.1,0.1,0.1)	0.050	0.052	0.052	0.049
TN(*s *= 20,30,40, μ = 0.5, 0.5, 0.5, σ^2 ^= 0.1,0.2,0.3)	0.047	0.054	0.051	0.043

**Table 2:** Empirical power at significance level 0.05 with 10000 replicates.

Distribution (sample sizes, parameters)	ANOVA	median	Welch	KW
Beta (*s *= 30, 30,30, *a *= 5,5,5,*b *= 20,25,30	**0.821**	0.576	0.810	0.775
Beta(*s *= 30, 30,30, *a *= 1.5,2,2.5, *b *= 20,20,20	0.650	0.504	0.648	**0.710**
Beta (*s *= 30, 30,30, *a *= 20,20,20, *b *= 1.5,2,2.5,	0.658	0.495	0.656	**0.713**
Beta (*s *= 20,30,40, *a *= 5,5,5, *b *= 20,25,30)	**0.792**	0.546	0.740	0.735
Beta (*s *= 20,30,40, *a *= 1.5,2,2.5, *b *= 20,20,20)	0.599	0.479	0.634	**0.670**
Beta (*s *= 20,30,40, *a *= 20,20,20, *b *= 1.5,2,2.5)	0.607	0.475	0.637	**0.665**
TN (*s *= 30, 30,30, μ = 0.45,0.5,0.55, σ^2 ^= 0.2)	**0.383**	0.240	0.378	0.362
TN (*s *= 30, 30,30, μ = 0.45,0.5,0.55, σ^2 ^= 0.1,0.2,0.3)	0.338	0.325	**0.412**	0.341
TN (*s *= 20,30,40, μ = 0.45,0.5,0.55, σ^2 ^= 0.2)	**0.349**	0.238	0.343	0.328
TN (*s *= 20,30,40, μ = 0.45,0.5,0.55, σ^2 ^= 0.1,0.2,0.3)	0.219	0.361	**0.423**	0.259

### A real data set

We will use a real methylation data set, the United Kingdom Ovarian Cancer Population Study (UKOPS) [15] with 274 controls, 131 pre-treatment cases, and 135 post treatment cases, to compare the performance of the proposed test with others. Those methylation data were generated by the Illumina Infinium Huamn Methylaytion27 BeadChip and can be downloaded under accession number GSE19711 from the NCBI Gene Expression Omnibus (http://www.ncbi.nlm.nih.gov/geo).

For this data set, there are 27578 loci. After data quality control process, we removed some subjects with BS values less than 4000 or the coverage rates less than 95%. We also separate subjects into 6 age groups (50-55, 55-60, 60-65, 65-70, 70-75, and 75 and over). Table 3 gives the numbers of subjects in each age by treatment groups. For each locus, we perform the above mentioned approaches.

**Table 3:** Number of samples in age group by treatment group used in the paper after removing subjects with bs <4000 or coverage rate <95% or age >80.

Age group	control	Pre-treat	Post-treat	Total
50_55	14	15	16	45
55_60	61	17	25	103
60_65	64	17	22	103
65_70	35	17	21	73
70_75	63	24	22	109
75_over	20	18	9	47
Total	257	108	115	480

## Results

### Simulation results

Table 1 reports the estimated type I error rates from each method under different conditions. For most of the time, the estimated type I error rates are close to the nominal significance level as expected. Table 2 gives the empirical powers from each method. It can be seen that the non-parametric method of Mood's median test usually has the lowest powers in the simulations. None of the ANOVA, Welch and KW tests is uniformly most powerful. In words, their performances depend on the distributions from which the data are generated. From our simulation study, the KW test is usually as powerful as or more powerful than the ANOVA test. The true distributions of the *β *-value may vary from locus to locus; it is impossible to simulate all possible distributions. However, based on the observation of the real data, we know that the distributions of the *β *-value are far from the normal distribution, under which ANOVA is the best test. Therefore, we prefer nonparametric tests which are more robust.

### Results from real data set

**Figure 1:**
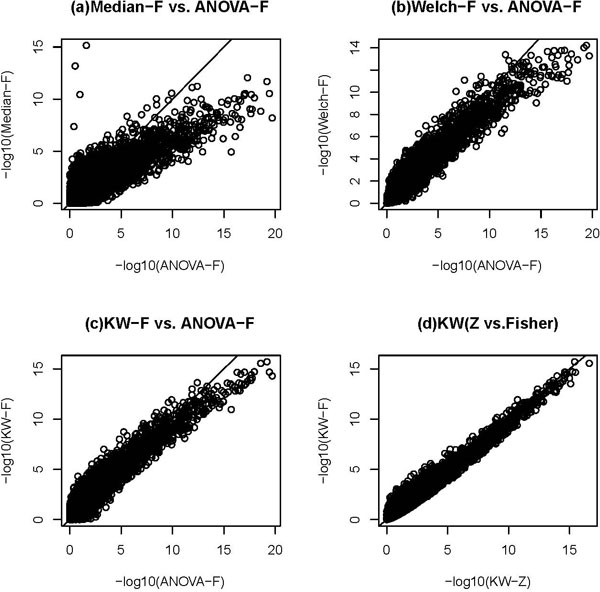
**Negative log10(p-value) from pair of methods**. Negative log10 p-values from pair of methods. (a) Combined ANOVA test vs. combined median test both using Fisher methods to combine p-values. (b) Combined ANOVA test vs. combined Welch test both using Fisher methods to combine p-values. (c) Combined ANOVA test vs. combined Kruskal-Willas test both using Fisher methods to combine p-values. (d) Combined KW test using Z test vs. combined KW test using Fisher to combine p-values.

For the real data set, we applied the above mentioned methods to get the overall p-values (either using Fisher or Z test to combine p-values from individual age groups) for each locus. Then we use various cutoff p-values, 0.001, 0.0001, 0.00001, and 0.000001, to count how many loci have smaller p-values for each method. Table 4 reports the results. We can see that the KW method usually finds more significant loci than other methods. It also shows that the two combining p-value methods, Fisher and Z test have similar performances, although Z test usually give a little bit more significant loci expect for the Median test. Figure 1 plots the negative log10 p-values from pairs of the methods. It shows that the KW method gives smaller p-values especially when the differences among the three treatment groups are not large (e.g., the negative log10 p-values between 3 and 6). From Figure 1 we can see that for a given cutoff p-value, most of the loci identified by ANOVA test or Median were also detected by the Welch test; in turn, most of the loci identified by Welch test were also detected by the KW test. This indicates the KW test is more powerful than other methods compared.

**Table 4:** Number of significant differentially methylated loci detected for given cutoff p-value based on the real data.

Method	1e-3		1e-4		1e-5		1e-6	
	
	Fisher	Z-test	Fisher	Z-test	Fisher	Z-test	Fisher	Z-test
ANOVA	981	1079	655	690	479	499	350	375
Median	906	893	464	449	255	240	143	127
Welch	1096	1106	640	673	416	424	281	289
K-W	**1359**	**1340**	**823**	**859**	**551**	**590**	**381**	**401**

## Discussion and conclusions

Due to the unique feature of the *β *-value of methylation data, traditional statistical methods, such as linear regression and ANOVA test may not be appropriate. It has been shown that methylation is highly correlated with age; ignoring age effect may cause many false positives and/or false negatives. The effect of age may also not be linear; therefore we need a better way to account for this effect. In this paper, we use p-value combination method to deal with age effect. For each age group, we use nonparametric method to compare the treatment groups. It is important to find powerful and robust nonparametric methods for this sort of data. Although we found that KW method is more powerful than some other nonparametric methods for methylation data, it is desirable to find more powerful tests in this area. Furthermore, we want to point out that there are many other methods can be used to combine p-values [22,23]; it may also be possible to find a more powerful method to combine p-values for Illumina Array Methylation data. However, based on our experiences, Fisher test is more robust and can be used in situations when a small portion of the p-values are very small; while the Z test is more powerful when the effect sizes are similar (e.g., the p-values don't differ much) for all of the age groups. Finally, although in this paper we use different cutoff p-values to compare the performance of tests, one may want to control the false positive rate. Several multiple comparison methods have been proposed for large scale data set to deal with the situations where the variables (loci) are not independent [24-28]. However, it remains to study which approach is more appropriate for the methylation data.

## Competing interests

The authors declare that they have no competing interests.

## Authors' contributions

ZC devised the basic idea of the new method and drafted the manuscript; HH, JL participated in study design data analysis; HKTN, SN, XH and YD assisted the study and co-wrote the manuscript. All authors read and approve the final manuscript.
